# ASO Author Reflections: Leave Policy for Breast Surgical Oncology Fellows: Finding the Path Forward

**DOI:** 10.1245/s10434-026-20344-6

**Published:** 2026-07-29

**Authors:** Melissa M. Rangel, Puneet Singh, Andrea Madrigrano, Heather A. Lillemoe, Helen M. Johnson

**Affiliations:** 1 Department of Breast Surgical Oncology, The University of Texas MD Anderson Cancer Center, Houston, USA; 2 General Surgery, Rush University Medical Center, Chicago, USA

## Past

During the rigors of surgical training, fellows are not immune to major life events such as childbirth, bereavement, illness, and caregiving. Historically, formal leave policies for many surgical fellowships have been lacking. Whereas the Accreditation Council for Graduate Medical Education (ACGME), the Fellowship Council, and the American Board of Medical Specialties have issued broad leave recommendations and several specialty boards have established their own policies, no analogous standard exists for breast surgical oncology (BSO) fellowship programs.^1–3^ Leave has instead been managed at the institutional level by fellowship leadership, with individual programs determining on a case-by-case basis whether to escalate a leave request to the Society of Surgical Oncology (SSO) and American Society of Breast Surgeons (ASBrS) Fellowship Training Committee for approval.

## Present

This study is the first national evaluation of leave policy and practice across accredited BSO fellowship programs.^4^ Through a survey of program directors achieving a 73% response rate and review of redacted Training Committee meeting minutes, the authors found that one third of programs lack a formal leave policy. Maximum leave duration varied widely between programs (median, 4 weeks; range, 0–12 weeks). When given, leave for non-birthing parents or bereavement was shorter than leave for birthing parents or medical purposes. The most commonly cited barrier to leave implementation was concern about completing fellowship requirements without extending training.

## Future

These findings underscore the need for a centralized leave framework that defines minimum leave entitlements, clarifies graduation requirements, and is accessible to trainees and program directors alike. The Fellowship Council’s model of 8 weeks maximum leave and 44 weeks minimum clinical activity in a 1-year fellowship may serve as a useful template.^1^ Notably, the BSO Fellowship Training Committee recently incorporated two flexible weeks into the revised fellowship curriculum, affording greater opportunity to accommodate leave without compromising program completion.^5^ Future work should capture trainee-level experiences and the financial implications of leave and training extension. A well-designed leave policy should work to preserve both educational rigor and trainee well-being.
